# Implementation of large language models in electronic health records

**DOI:** 10.1371/journal.pdig.0001141

**Published:** 2025-12-19

**Authors:** Maxime Griot, Jean Vanderdonckt, Demet Yuksel

**Affiliations:** 1 Cliniques Universitaires Saint-Luc, Woluwe-Saint-Lambert, Brussels, Belgium; 2 Université Catholique de Louvain, Louvain Research Institute in Management and Organizations, Louvain-la-Neuve, Wallonia, Belgium; 3 Université Catholique de Louvain, Institute of NeuroScience, Woluwe-Saint-Lambert, Brussels, Belgium; Australian Institute of Health Innovation, AUSTRALIA

## Abstract

Electronic Health Records (EHRs) have improved access to patient information but substantially increased clinicians’ documentation workload. Large Language Models (LLMs) offer a potential means to reduce this burden, yet real-world deployments in live hospital systems remain limited. We implemented a secure, GDPR-compliant, on-premises LLM assistant integrated into the Epic EHR at a European university hospital. The system uses Qwen3-235B with Retrieval Augmented Generation to deliver context-aware answers drawing on structured patient data, internal and regional clinical documents, and medical literature. A one-month pilot with 28 physicians across nine specialties demonstrated high engagement, with 64% of participants using the assistant daily and generating 482 multi-turn conversations. The most common tasks were summarization, information retrieval, and note drafting, which together accounted for over 70% of interactions. Following the pilot, the system was deployed hospital-wide and adopted by 1,028 users who generated 14,910 conversations over five months, with more than half of clinicians using it at least weekly. Usage remained concentrated on information access and documentation support, indicating stable incorporation into everyday clinical workflows. Feedback volume decreased compared with the pilot, suggesting that routine use diminishes voluntary reporting and underscoring the need for complementary automated monitoring strategies. These findings demonstrate that large-scale integration of LLMs into clinical environments is technically feasible and can achieve sustained use when embedded directly within EHR workflows and governed by strong privacy safeguards. The observed patterns of engagement show that such systems can deliver consistent value in information retrieval and documentation, providing a replicable model for responsible clinical AI deployment.

## Author summary

We built and deployed an artificial intelligence assistant that helps clinicians find and summarize information directly inside the hospital’s electronic record system. Our goal was to reduce the time clinicians spend searching through long medical files and writing notes, allowing them to focus more on their patients. The system runs entirely within the hospital’s secure network to protect patient privacy and meets European data protection standards. We first tested the assistant for one month with a small group of clinicians to see how they would use it in daily practice. After positive feedback, it was made available to all hospital staff. Over five months, more than one thousand users used it, mostly to look up information or prepare summaries of patient records. Our work shows that large language models can be safely integrated into real hospital systems when strong safeguards and clear oversight are in place. Our approach could serve as a model for other healthcare institutions.

## Introduction

The digitization of the medical field brought many benefits, such as better continuity of care, centralized access to data, and larger volumes of data for research [1,2]. However, these benefits are not without cost; physicians spend more time documenting since the introduction of Electronic Health Records (EHRs), with some spending more than 50% of their time using EHRs [3,4]. This additional time spent is partially explained by the need to document the same information in different formats. Previously, clinicians documented diagnoses only in clinical notes; today they must also enter them into structured EHR fields, effectively duplicating the task [5]. This results in either increased documentation time and dissatisfaction or incomplete documentation, often in structured fields. In addition to this double documentation burden, the availability of clinical documents also increases the volume of information available that physicians have to process when preparing a consultation, on average, 20 minutes per patient for emergency physicians at our hospital. This dual documentation burden increases clinician stress and reduces the time available for patient care [6]. Even though EHR systems offer features to facilitate conversion to structured data, many aspects of optimal EHR usage remain dependent on individual behaviors and cannot be fully controlled.

LLMs have garnered much attention in medicine, with models achieving impressive scores on medical benchmarks [7]. Their impressive ability to process large volumes of text was rapidly identified as a potential solution to handle medical tasks. However, most research focuses on support for clinical reasoning, targeting diagnostics or treatment planning on synthetic benchmarks [8].

Despite these promising results, translating such capabilities into real clinical practice remains a major challenge. Medicine is a critical field, making it difficult to access real-world clinical data and to perform real-world experiments. As a result, AI research often diverges from real clinical conditions. The developed assessment tools have been largely based on multiple choice questions (MCQs) used to evaluate medical students and residents [9]. While the relevance of MCQs to evaluate LLMs is debated [10,11], they have the benefit of being reproducible, easy to administer, and most importantly available [12–14]. While the importance of testing in the real world was emphasized in medical literature, investigations have been lacking, with most real-world evaluations being limited to specific tasks in controlled environments [15]. The two common approaches for real-world clinical evaluation are either based on generating or transforming existing textual data, or generating textual data from speech data, including ambient listening.

### Text-to-text

Evaluations of LLMs in clinical workflows have generally addressed isolated tasks and rarely examined performance under real-world conditions. Decker et al. [16] showed that LLM-generated informed-consent documents for common surgical procedures were comparable to, and occasionally surpassed, those written by surgeons. Zaretsky et al. [17] likewise demonstrated that LLMs can create patient-friendly discharge summaries of acceptable quality. Automatic drafting of patient-message replies has also been piloted in Epic, but Tai-Seale et al. [18] found no measurable reduction in workload. Baxter et al. [19] examined retrospective clinical notes and reported that LLM-generated drafts often required substantial revision, particularly for messages communicating unfavorable information.

Beyond document generation, recent evidence shows that LLMs can perform clinically meaningful diagnostic reasoning on real-world data. Vrdoljak et al. [20] conducted a prospective study using 73 authentic emergency internal-medicine cases and found that OpenAI’s o1 model achieved human-level diagnostic accuracy (97.3 %) and statistically indistinguishable overall quality ratings compared with physicians, while smaller models such as Claude-3.5-Sonnet and Llama-3.2 70B underperformed mainly in therapy planning. These findings complement prior randomized and observational evaluations showing that LLMs can support or even rival clinicians in diagnostic reasoning, though gains depend strongly on model scale and post-training reinforcement [21–23]. Overall, current models show measurable diagnostic competence on real patient data, yet rigorous validation and governance remain prerequisites for deployment.

A recent scoping review of clinical summarization studies by Bednarczyk et al. [24] identified only retrospective investigations that relied on publicly available datasets, a limitation that restricts analyses to a narrow set of medical specialties. Although study methodologies varied, most focused on output quality and error characterization rather than on effects within clinical workflows. To promote more consistent assessment, Asgari et al. [25] introduced an annotation framework that enables fine-grained error reporting and showed that hallucination and omission rates can approach or even fall below those of human experts. Complementing this, MedHELM by Bedi et al. [26] offers a comprehensive taxonomy spanning five categories, 22 sub-categories, and 121 clinical text tasks. Their benchmark suite indicates that current LLMs achieve the strongest performance in clinical-note generation and patient communication, findings that align with the individual task studies discussed above. Nevertheless, these benchmarking efforts, like earlier studies, do not quantify the downstream impact on clinical workflows.

### Speech-to-text

Another branch of real-world evaluation involves generating clinical text from speech data with ambient listening tools that listen to clinician-patient conversations and draft documentation. These systems typically combine automatic speech recognition with LLM-based summarization to produce clinical notes, aiming to relieve physicians from manual charting and allow more focus on the patient. Early results have been encouraging. For instance, Balloch et al. demonstrated in a controlled simulation that an AI “ambient scribe” could produce higher-quality outpatient documentation (measured by standard scoring) while also shortening visit lengths by about 26% without sacrificing patient interaction time [27]. Clinicians in that study reported a reduced cognitive burden and a better overall experience when the AI handled note-taking. In real clinical settings, preliminary deployments have shown similar promise. A pilot implementation of an ambient AI assistant in primary care found that automatically generated draft notes significantly decreased the time physicians spent on after-hours charting and improved their work satisfaction [28]. On a larger scale, Kaiser Permanente recently rolled out an ambient AI scribe to over 7,000 physicians across 17 medical centers, reporting an estimated 15,700 total hours saved in documentation (about 1 minute less EHR time per patient visit) along with substantially improved physician-patient engagement and job satisfaction [29]. Notably, 84% of surveyed doctors in that deployment felt the AI system allowed them to connect more with patients, and patient feedback was predominantly neutral or positive regarding the tool’s presence.

Qualitative evaluations further underscore these benefits. In interviews, physicians have described ambient AI scribes as having a positive impact on their workload, work-life integration, and even patient engagement during visits [30]. In practice, current ambient documentation tools still require clinicians to review and edit the AI-generated notes, and no studies yet quantify their effect on clinical outcomes or error rates. In summary, speech-driven LLM tools offer a compelling solution to documentation burdens and have shown improvements in efficiency and provider satisfaction, but rigorous real-world validation—especially regarding quality assurance and workflow impact—remains an active research area.

### Contribution

Most research emphasizes accuracy on synthetic benchmarks, but little is known about how to deploy and integrate LLMs in real clinical environments. Moving from controlled evaluations to live systems introduces challenges in security, governance, adoption, and workflow integration that current literature rarely addresses. This study addresses these implementation challenges rather than efficacy measurement, focusing on the technical architecture, security framework, and real-world adoption patterns of LLM deployment in clinical practice. Rather than measuring clinical outcomes, we evaluate whether physicians will adopt LLM tools when properly integrated into existing EHR workflows and characterize how they naturally use such systems in practice.

The main contributions of this work are as follows:

- We propose a comprehensive security and governance framework for deploying clinical artificial intelligence systems, emphasizing GDPR compliance, on-premises hosting, and institutional oversight.
- We present a full technical implementation of a large language model integrated directly into a live electronic health record environment, providing a replicable architecture for secure clinical deployment.
- We report empirical evidence from a one-month pilot involving 28 physicians across nine specialties, with 64% using the system daily during real-world testing.
- We extend these findings to hospital-wide production use, documenting adoption by 1,028 users who generated 14,910 conversations over five months, confirming sustained engagement and scalability.
- We analyze usage patterns to show how clinicians naturally interact with language-based assistants, with predominant use for information synthesis and retrieval rather than diagnostic reasoning.

## Materials and methods

The goal of the system is to integrate seamlessly into the day-to-day workflow of physicians. We held early workshops to familiarize physicians with LLMs and to collect their envisioned use cases [31]. Through this process, we obtained a set of use cases prioritized by the physicians themselves. The most requested use cases were related to improving access to information, be it patient information, scientific literature, or internal procedures.

A chatbot that can retrieve, summarize, and interact with the different sources of information could address most of the use cases proposed by users and serve as a generic entry for current and future use cases. We identified three axes to ensure the tool’s usage does not become an additional burden to physicians:

1. The Electronic Health Record must be the initiator and host of the application to reduce context switches and centralize the information in a single application
2. The user interface must be familiar and simple enough to be used without additional training. As users are increasingly used to tools such as ChatGPT, a similar interface appears optimal
3. The integration must not get in the way of existing workflows and should only appear on demand

### Integration

Since 2020, the Cliniques universitaires Saint-Luc hospital has utilized the Epic EHR system, which offers various mechanisms for integrating third-party applications. We selected web-based integration, as Epic can embed web apps directly in its interface and provide secure access via the SMART on FHIR protocol.

This study is designed as a deployment feasibility and adoption study rather than a clinical efficacy trial. Our primary objectives are to demonstrate technical implementation, measure user adoption, and characterize usage patterns. We deliberately did not measure clinical outcomes or time savings, as this would require a controlled study design inappropriate for an initial deployment evaluation.

#### Infrastructure.

LLMs require extensive computing capabilities, especially to support thousands of users in a university hospital. The GenAI strategy of our institution is to host medical applications on-site and not to rely on vendor services that do not allow for the level of customization and validation required to make these tools useful and safe in day-to-day workflow.

To support our GenAI efforts, we invested in a server with 8 NVIDIA H200 GPUs, allowing us to host the largest available models, such as DeepSeek V3. In addition to our inference capabilities, this server provides the necessary compute to fine-tune small to medium-sized models on specific tasks.

Although the institutional server provides ample computational power, its cost ($400,000) makes it inaccessible for most hospitals. Importantly, this infrastructure serves multiple purposes beyond LLM inference for the assistant—such as automated clinical coding, model training, and internal research projects—thereby distributing its cost across several initiatives. For inference-only deployments, smaller models now achieve comparable performance for many clinical information retrieval and summarization tasks [32]. These lighter models can be hosted on far more modest hardware, with acquisition or rental costs under $50,000, provided that equivalent privacy and security guarantees are ensured. Such configurations offer a feasible pathway for institutions seeking scalable and affordable implementations without compromising data governance [33].

**Fig 1:**
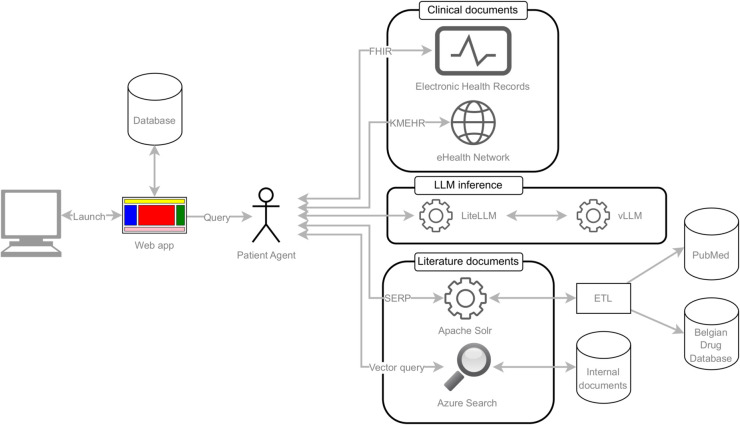
System components. Overview of the software architecture and information flow between the components.

Our software stack is depicted in Fig 1 based around vLLM [34], which provides the LLM inference capabilities using the same protocol as OpenAI, allowing us to use existing libraries and methods to interact with the LLM. Performance evaluation showed that the server sustains a throughput of approximately two requests per second under concurrent load, with batch processing enabling up to 50 requests to be handled simultaneously. This capacity is well above the expected demand for routine clinical use and further supports the feasibility of smaller-scale deployments.

#### Cognitive engine.

We deploy Qwen3-235B in FP8 precision [35], enabling efficient inference at scale without compromising model quality. To ensure generated outputs adhere to expected formats, we use vLLM’s structured decoding and tool use features. Structured decoding constrains the model to produce outputs that follow predefined schemas, which is critical for downstream systems that rely on predictable, well-formed inputs.

We use LiteLLM to manage access control and system reliability by enforcing usage limits and distributing requests across multiple vLLM instances. This allows us to maintain stable performance and fair resource allocation, even under heavy demand.

### Retrieval augmented generation

**Fig 2:**
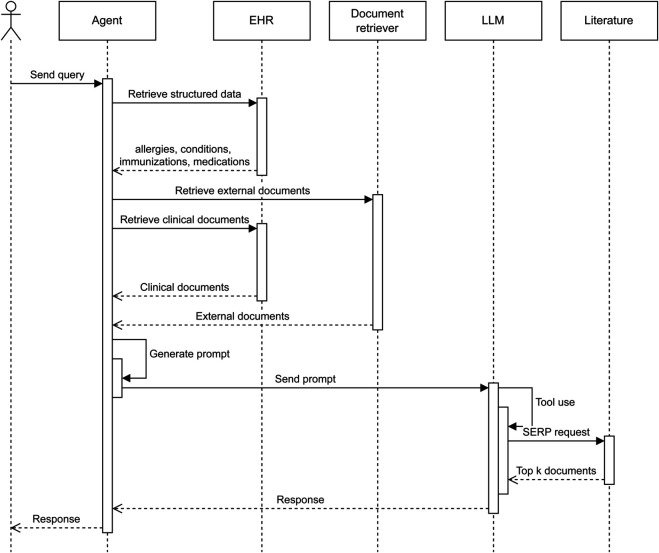
Response generation workflow. UML-based overview of the end-to-end process from user query to system-generated response.

LLMs are powerful tools, but their effectiveness is limited by the scope of their training data and the content available in their inference context. To improve relevance during inference, it is necessary to inject additional knowledge through Retrieval Augmented Generation (RAG) as depicted in Fig 2. This can be done through two main strategies: **passive injection**, where a fixed set of knowledge is always included, and **active injection**, where relevant data is retrieved dynamically based on decisions made by the model or the user.

We also distinguish between internal and external knowledge sources. For external sources that pose a risk of data leakage, such as full-text search engines, we apply an Extract-Transform-Load (ETL) process to create a local copy of the data. This process formats and indexes content in a controlled environment and filters it to retain only curated, relevant subsets.

The core inference and retrieval pipeline operates entirely within the hospital’s internal network and does not require outbound internet connectivity. Access to Microsoft SharePoint documents hosted on a private Azure tenant is mediated through a secured institutional gateway and restricted to non-patient administrative content. No patient data are transmitted outside the hospital network as part of this process

#### Common knowledge.

Given the large context windows of current models, we always prepend essential background information to the prompt. Specifically, we include the current date, hospital location, and the provider’s name and role. We then add patient data extracted from structured EHR fields via FHIR: demographics (sex, age, encounter type, location) and clinical data (active allergies, chronic conditions, medications, and immunizations). Each clinical datum is accompanied by its recording date, enabling the model to detect and, when necessary, question outdated information.

#### Internal clinical documents.

FHIR exposes several document resources stored in the EHR, such as clinical notes and radiology reports. This includes documents imported from our historic EHR. For every user query, we retrieve the complete, chronologically ordered list of signed documents and filter out those addressed to the patient or with low information density. We select up to 20 documents and append their content to the prompt until we reach a 50,000-character budget, remaining safely within the model’s context window. For each document, we supply its title together with the signature date.

#### External clinical documents.

Belgium’s eHealth platform employs a decentralized architecture for sharing healthcare documents. Because our hospital is located in Brussels, we use the *Réseau Santé Bruxellois* (RSB), one of the platform’s four regional networks. Through RSB, care providers exchange documents (examination results, clinical notes, correspondence) via web services that implement the KMEHR protocol [36]. To ensure we did not overload the eHealth platform, we filtered documents by type and retained only Discharge letters, Consultations, and Imaging reports. After filtering, we selected only the top 10 most recent documents. This decision was made based on the assumption that discharge letters are written to contain all the necessary information for the clinicians to understand the patient’s medical history.

Unlike the internal corpus, external files are heterogeneous and often stored as PDFs, whose reliable parsing is non-trivial. We therefore rely on Docling [37], an open-source, fully on-premise tool that converts numerous formats (including PDF) into machine-readable representations such as HTML or Markdown (additional details on implementation are available in S1 Appendix).

#### Internal literature.

The hospital maintains an internal Microsoft SharePoint in which procedures, guidelines, and other local documentation are stored. This collection is heterogeneous, mostly containing complex PDF and Word files in French. These documents are indexed in an Azure Search Service, which is a vector database used to retrieve documents by passing the embedding vector of the search query.

The major challenge of leveraging the internal documentation is the extraction of documents. PDF files, for instance, are complex to process, especially in non-English languages. The documents also contain many images and tables that are not extracted, further complicating the extraction process. Considering this internal documentation is incomplete and secondary during clinical practice, we did not perform extensive configuration or experiments beyond the standard extraction and vectorization pipeline offered by Microsoft.

#### External literature.

Evidence-based medicine is the standard of care, but staying up to date and recalling the details of guidelines is challenging. Physicians often rely on external documents that they review to make optimal decisions for patient care. However, this process is often time-consuming as physicians must find the **relevant documents**, find the **relevant information** in complex documents that can contain hundreds of pages, and often **leave the EHR** to access these documents. The goal of integrating external literature is therefore to address these three concerns to speed up access to the relevant information.

Contrary to the internal literature, which is heterogeneous and difficult to parse, the external documents are homogeneous and structured. This allowed us to use a different approach, not based on vector databases but on classical text search in the content and the metadata. This gives the model the ability to generate different queries and improve the queries based on the result list.

Identifying relevant documents in the exponentially growing scientific literature is a challenging task in itself, so much so that physicians often rely on private databases such as UpToDate [38] that synthesize medical literature. However, these databases do not allow automatic processing, which implies that alternatives must be found. We therefore rely on the suggestions of physicians made during the workshops [31] and identify subsets of PubMed to reduce the search space and improve the likelihood of finding relevant documents.

Contrary to internal literature, the well-formed and structured nature of the documents allows for more accurate searches, especially semantic. We therefore opted for full-text semantic search using Apache Solr instead of vector databases. The documents are chunked based on level 1 and 2 titles present in the documents.

The LLM uses a tool call to make a query to Apache Solr with a full-text query, and the documents and their contents are then returned. We make an additional call to the LLM with a list of document names and ask the LLM to pick the top 3 documents, acting as a ReRanker. These 3 documents are then injected as a tool call result.

#### Integrated in the EHR.

Considering we select the documents and sources in advance, we have a comprehensive list of valid URLs allowed to be opened directly in the EHR, which we whitelisted. As detailed in the User Interface section, the documents used by the LLM are listed and displayed as clickable links to open the source of information in a tab directly in the EHR, ensuring quick access to the original documents.

### User interface

Physicians are increasingly familiar with conversational AI tools such as ChatGPT and Claude, which typically employ a minimalist chat-based interface accompanied by a list of previous interactions. We built on the open-source OpenWebUI interface, simplifying it for clinical users while preserving its core chat functionality [39].

**Fig 3:**
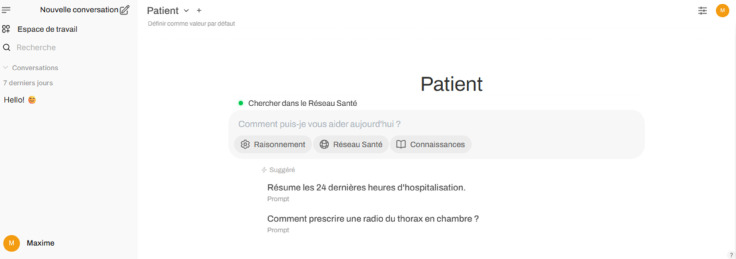
User interface. Screenshot of the EHR integrated interface with the conversation history panel (left) and the central chat workspace, with toggle buttons for reasoning, the RSB, and knowledge bases.

Originally designed for advanced users seeking fine-grained control over language models, OpenWebUI includes numerous configuration options. To streamline the interface for clinical use, we removed most advanced features and retained only the essential interaction loop. In addition, we introduced several enhancements to support day-to-day clinical workflows as shown in Fig 3, including:

1. **Reasoning control**, which allows users to toggle between a standard and a faster non-reasoning mode;
2. **Source control**, enabling clinicians to specify which data sources should contribute to the model’s response;
3. **Data retrieval status**, providing visibility into the documents retrieved by the system;
4. **Scoped conversations**, which help maintain contextually relevant and patient-specific interactions.

#### Explainability.

**Fig 4:**
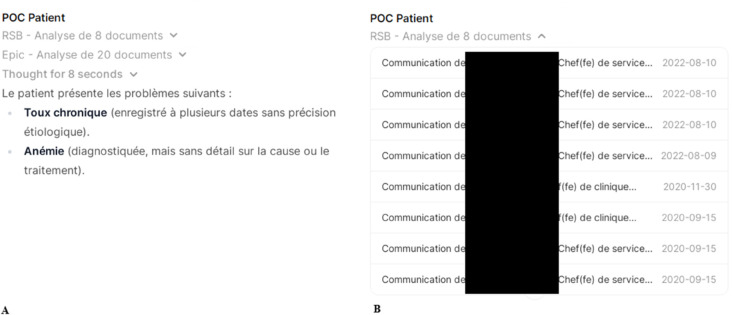
Source references. User interface showing the assistant’s response with collapsed document sources in panel A, and the expanded RSB sources in panel B showing the title of the documents used and their publication date. In panel B, the names of physicians are hidden for privacy reasons.

In a medical context, ensuring the correctness of information is not just desirable; it is mandatory. To support this, our system emphasizes transparency throughout the retrieval and response generation process. Every answer generated by the model is accompanied by a structured list of the sources consulted, as shown in Fig 4A and 4B. These sources include both documents within the EHR and external clinical references. For external documents, the system provides direct hyperlinks that open the relevant material in a dedicated EHR-integrated reading window, allowing physicians to review the original context without leaving the clinical interface. This design not only facilitates rapid verification but also reinforces trust in the AI’s recommendations by enabling traceability and auditability of every response.

### Security, privacy, and governance

The deployment of generative AI tools in clinical settings requires not only technical robustness but also a governance model that ensures safety, accountability, and compliance with legal and institutional policies. Our governance framework was developed in collaboration with medical leadership, the legal department, cybersecurity teams, and frontline clinicians to ensure responsible innovation.

#### Institutional oversight and ethics.

The ethics committee classified the project as a practice study, allowing live deployment without a formal clinical trial protocol. This classification allowed for iterative development while maintaining alignment with ethical oversight standards.

The hospital’s medical leadership was engaged early in the process, and a collective decision by the medical direction authorized the system’s deployment in production. The tool was designed to be non-interventional, fully optional, and integrated into existing workflows without altering clinical responsibilities or decision-making.

A key component of governance was the involvement of the hospital’s network of physician “EHR champions,” representing multiple specialties. These clinicians participated throughout the development lifecycle, from use-case definition to interface testing. Before go-live, the application was demonstrated in a test environment during a dedicated one-hour review session, which served to surface potential limitations and incorporate clinician feedback into final refinements.

In parallel, the hospital’s Data Protection Officer (DPO) was consulted to ensure compliance with privacy and data protection regulations. A Data Protection Impact Assessment (DPIA) was completed before deployment, addressing potential risks associated with the use of sensitive health data and documenting mitigation strategies. The DPIA also confirmed that no patient data would leave the hospital infrastructure, and that the application met the standards of the EU General Data Protection Regulation (GDPR) [40].

#### Access control and data minimization.

Access control is enforced through the SMART on FHIR launch protocol, which delegates user identification and authorization to the EHR system. When a user launches the application, Epic issues a context-specific access token that scopes the data the application can retrieve based on the user’s role, the selected patient, and the configured application permissions.

This approach ensures strict adherence to the principle of data minimization: the system can only access information that the user is already authorized to view within the EHR interface. No persistent data storage occurs outside of the application’s runtime session, and all access to patient data is ephemeral and auditable.

By leveraging Epic’s access controls, the system inherits existing institutional safeguards, such as role-based access restrictions and user audit logging. This design ensures that data retrieval remains compliant with internal policies and external regulations while minimizing the surface area for potential misuse.

#### Model governance and updates.

All models deployed in this environment are hosted on-premise and are subject to internal validation procedures. Before updating or fine-tuning a model, we evaluate it on MedQA [12] and internal standardized tasks in test environments.

A formal versioning system is maintained to ensure traceability of responses to specific model builds. Changes and new models are rolled out to champions who validate the models in real clinical contexts for a fixed period of time, depending on the magnitude of the change. Based on their feedback, the model is then pushed to all users or rejected.

#### Compliance with legal and regulatory frameworks.

The system was notified to the Belgian Federal Agency for Medicines and Health Products as an *in-house medical device* under Article 5(5) of the European Medical Device Regulation (MDR) [41]. This provision permits healthcare institutions to develop and use custom medical devices internally, provided that applicable requirements for safety, performance, and quality management are met. Based on its intended clinical functionality and risk profile, the system corresponds to a class IIa medical device under the MDR classification rules.

With respect to data protection, the system was designed in full compliance with the General Data Protection Regulation (GDPR) [40]. No personal data leave the hospital’s infrastructure, and access to patient information is strictly limited to what is required for the user’s current clinical context. All data access is enforced through role-based permissions managed by the EHR and is subject to institutional audit logging.

The European Union Artificial Intelligence Act (AI Act) entered into force in 2024, with several obligations for high-risk AI systems being phased in over time [42]. Given the clinical context of use, we proactively aligned the system’s development and governance with the requirements applicable to high-risk medical AI systems. In particular, we implemented:

- Clear documentation of intended use, model behavior, and known limitations;
- Comprehensive technical documentation of the system architecture and data pipelines;
- Internal traceability and version control for all model updates and configuration changes;
- User training materials and interface safeguards to mitigate inappropriate reliance on the system;
- Organizational policies supporting transparency, accountability, and post-deployment monitoring.

Together, these measures ensure compliance with current European medical device and data protection regulations and position the system to meet the obligations of the AI Act as they become fully applicable.

#### Incident management and user feedback.

To support safe and responsible deployment, a real-time feedback mechanism was integrated directly into the user interface. Clinicians can rate each response on a scale from 1 to 10, select one or more predefined reasons for their score (e.g., hallucination, irrelevance, omission), and optionally provide free-text comments to elaborate on their evaluation.

Submitted feedback is logged and reviewed by the project team, with reported incidents triaged on a rolling basis and systematically reviewed in monthly meetings. Critical issues—such as incorrect clinical reasoning or information retrieval failures—are prioritized for investigation and resolution.

In addition, a user feedback dashboard aggregates both quantitative metrics (e.g., usage frequency, latency, error rates) and qualitative data (e.g., user comments, flagged examples). This enables continuous monitoring of system performance and user satisfaction.

This physician-in-the-loop feedback loop supports iterative refinement of the system and ensures that future development is guided by clinical needs, usability concerns, and real-world performance.

## Results

The one-month pilot study enrolled 28 senior attending physicians from nine specialties: oncology, geriatrics, internal medicine, pediatrics, intensive care (totaling 35,354 hospitalizations in 2024), surgery (20,626 interventions in 2024), emergency medicine (79,506 visits in 2024), ophthalmology, and radiotherapy (2 linear particle accelerators and 2 tomotherapy machines). The intervention focused exclusively on physicians’ practice patterns; no patient-level clinical outcomes were collected. During this one-month pilot, we initially only enabled internal clinical document retrieval to allow clinicians to gain experience with the system without the additional complexity of understanding data flow. During the last week of the pilot, the complete system was enabled.

### Adoption

After completing the brief training session, physicians received production access to the model. Over the 30-day observation window, 18 physicians interacted with the system daily, whereas 5 discontinued use after their initial exploration. Adoption remained high despite technical issues during the first week (e.g., a missing launch icon for certain patients and occasional failures to retrieve clinical documents). In total, 482 conversations, 1630 messages were logged, and 47 discrete feedback items were collected: 25 positive and 22 negative. Analysis of interaction timestamps showed consistent daily engagement among active physicians, who averaged 2.32 conversations per day (median = 2.0, IQR = 1.17).

Radiotherapy clinicians quickly developed optimized prompts and shared them informally to speed up information retrieval. This behavior suggests that the high engagement observed may be partially attributable to the curiosity and technological enthusiasm of the volunteer cohort.

### Usage patterns

Automated classification using the Qwen3-235B model (source available in S1 File) of the first user message and the automatically generated conversation title (without inspecting model responses or subsequent turns to preserve patient anonymity) revealed four predominant usage patterns:

- **Summarization**
- **Information retrieval**
- **Differential diagnosis**
- **Note writing**

As conversations often involved several exchanges, their purpose sometimes shifted between use cases. However, we report only the initial message’s intent. Only five instances fell outside these four patterns: one asking for a drug reimbursement protocol, another translating a document, and three malformed queries. This concentration of usage in four patterns can be explained by the recent introduction of the tool, which did not allow users enough time to become confident enough with the tool to test different kinds of approaches.

#### Summarization.

Summarization was the most common workflow, representing 29.5% of all uses (*n* = 142). Emergency department physicians, in particular, used the model to prepare consultations. The ability to process dozens of documents concurrently enabled rapid assimilation of a patient’s history. Only one hallucination was reported: the model indicated that the patient was being considered for palliative care, which was absent from the record. The attending physician noted, however, that this was a true clinical consideration that had not yet been documented. Four omission events were also reported, with only one having details on the omission, which was related to pathology results that were not correctly indexed in our FHIR implementation.

#### Information retrieval.

Targeted information retrieval ranked second in frequency with 29.2% of uses (*n* = 141). Typical queries included requests for results of prior investigations, family history, or medication dosages. Geriatricians additionally retrieved variables needed for risk scores such as HEMORR2HAGES [43]. While the model accurately surfaced the raw data, it misinterpreted certain score components; for example, it failed to recognize that the bleeding-risk element of the HEMORR2HAGES score refers specifically to active bleeding.

#### Differential diagnosis.

The model was also consulted for differential diagnosis in 25.1% of cases (*n* = 121). In one illustrative exchange, a physician queried: “Can you find an explanation for the desaturation?” The system returned several possibilities and prompted consideration of tuberculosis, an option the team had not initially considered. Overall, diagnostic support was less prevalent than retrieval or clerical assistance, aligning with prior expectations that LLMs have more potential as efficient data retrievers rather than decision makers.

#### Note writing.

Finally, physicians also leveraged the system to draft note sections based on chart data and their shorthand observations in 15.1% of cases (*n* = 73). This behavior is consistent with prior studies underscoring documentation burden in clinical workflows [3]. No feedback was provided for this use case.

### Feedback

During the study, 815 assistant turns were eligible for rating; users reacted to only 47 of them (5.8%). Among those 47 ratings, 25 were positive (✓, 53.2%) and 22 negative (55, 46.8%), giving an almost even split.

- *Structured reasons.* Less than half of all ratings (21/47, 44.6%) carried no predefined reason (“-” in Table 1). Reasons were omitted more often with positive votes (15/25, 60.0%) than negative ones (6/22, 27.2%).
- *Free-text comments.* 18 ratings (38.3%) contained a comment. 13 of these comments accompanied negative votes, underscoring that users tend to justify a down-vote but rarely a thumbs-up.
- *Reason distribution.* When a reason was given, the most frequent negative labels were *Omission* (6/16), *Other* (4/16), *Misunderstanding* (3/16), *Factual error* (2/16), and a single instance of *Hallucination* was recorded. Positive reasons were led by *Accurate information* (5/10), *Attention to detail* (2/10), *Thorough explanation* (2/10), and a single instance of *Creativity*.

**Table 1:** Complete export of the feedback received during this pilot. After giving a thumbs up or down to a response, the users are provided with a list of predetermined reasons to choose from, as well as a free-text form to add a comment or detail the reason if none of the options match the problem. The comments are translated from French.

Rating	Reason	Comment
✓	accurate_information	It’s excellent that it mentions the patient is Dutch-speaking.
✓	accurate_information	The treating ophthalmologist question needs to be explained more clearly in the prompt.
✓	accurate_information	A slight improvement in the accurate understanding of a medical case: this concerns a patient undergoing hormone therapy for breast cancer, specifically with tamoxifen. Tamoxifen strengthens the bones, thus reducing the risk of osteoporosis, unlike other hormone therapies. Therefore, the following suggestion is not relevant for this type of hormone therapy (it could remain as a general preventive measure, but not in relation to the hormone therapy): Osteoporosis prevention: Measurement of bone mineral density (T-score) before initiating long-term hormone therapy.
✓	accurate_information	-
✓	accurate_information	-
✓	attention_to_detail	-
✓	attention_to_detail	-
✗	factual_errors	the family doctor is missing
✗	factual errors	She received cisplatin
✗	hallucination	"palliative care considered" is not mentioned in clinical notes (that being said, it’s true).
✗	misunderstanding	Seems to confuse preventive and therapeutic anticoagulation (different dosages), and does not properly sequence the timeline nor seem to distinguish between “presence of an embolism/thrombosis” vs. “past anticoagulation.” In general, I think AI will often be used to trace a past treatment/event: perhaps for this type of question, it would be good to redirect the user to Epic tools designed for that purpose? (summary/life chronology)
✗	misunderstanding	The treating ophthalmologist = external ophthalmologist who referred the patient.
✗	misunderstanding	-
✗	omission	The patient’s tumor marker has been measured every 3 months for a very long time.
✗	omission	XY is the family doctor
✗	omission	-
✗	omission	The AI seems to have access to imaging reports, but not pathology? It would be very useful!
✗	omission	-
✗	omission	-
✗	other	Answer in Spanish
✗	other	-
✗	other	incomplete information
✗	other	No response after 3 minutes
✓	showcased creativity	Tuberculosis had not been considered. We will exclude it.
✓	thorough_explanation	
✓	thorough_explanation	
✓	-	!!! did not accurately transcribe the surgical protocol described in the letter dated [REDACTED]: “a recession of the right lateral rectus muscle by [REDACTED] mm and a resection of the right medial rectus muscle by [REDACTED] mm”; instead, it found this information: “Recurrence after surgery in [REDACTED] (recession and resection of the right lateral rectus).”
✓	-	-
✓	-	-
✓	-	-
✓	-	-
✓	-	-
✓	-	-
✗	-	The system is amazing, but it does not have access to oncologic history
✓	-	-
✓	-	-
✓	-	-
✗	-	Not what I asked
✗	-	-
✓	-	-
✓	-	-
✓	-	-
✗	-	-
✓	-	-
✗	-	-
✓	-	-
✗	-	The conclusion is correct: anticoagulation is needed, and close monitoring is essential due to a high risk of bleeding. However, the details of the hemorrhage score are not entirely accurate. The first H = renal and hepatic insufficiency. E = ethanol (alcohol). M = correct (malignant pathology). O = older age (elderly). R2 = bleeding risk, but this should be assessed based on active bleeding (look for free text in the medical record mentioning this or evidence of red blood cell transfusion - a loss of 2 g/dL acutely is the cut-off to consider). The second H = labile hypertension. Look for blood pressure spikes and episodes of hypotension in the record. A = correct. G = correct. E = excess falls (risk of falling); search for free text in the file. S = correct.

The pattern suggests that users intervened primarily when the assistant either omitted clinically relevant context (e.g. pathology reports, prior chemotherapy, explicit HEMORR2HAGES variables) or produced output that was off-topic (wrong language, excessive latency). True content errors, such as hallucinations or factual mistakes, were infrequently reported in user feedback; however, the low overall feedback rate limits conclusions about their true prevalence.

Several factors plausibly reduced the feedback rate:

1. **Workflow pressure:** Clinicians in busy wards have limited time; unless a response is conspicuously wrong or extraordinarily helpful, providing a rating competes with patient-care tasks.
2. **Low friction for silence:** The pilot did not gate progress on submitting feedback; the path of least resistance was to say nothing.
3. **Perceived adequacy:** When the assistant’s reply was simply “good enough,” clinicians had little incentive to spend extra clicks to endorse it. This produced an asymmetry in which users wrote detailed explanations for negative ratings yet stayed silent after positive ones.
4. **Interface cues:** Structured reasons required an extra click and comments an extra text entry; each additional action step measurably decreases response rates.

These observations suggest that future deployments should (i) streamline the rating UI, (ii) actively prompt for brief positive comments, and (iii) log implicit signals (e.g., time-to-next-question) as complementary quality indicators when explicit feedback is scarce.

### Production use

Following the pilot, the assistant was rolled out hospital-wide to physicians, nurses, and students. This section summarizes routine, at-scale activity and complements the pilot’s feasibility results.

From May 8 to October 13, 2025, **1,028 unique users** generated **14,910 conversations** comprising **20,225 user messages**. For engagement analyses, we defined an *active* cohort as users who engaged at least once a week during this window since their first interaction; **561 users (54.6%)** met this criterion.

Among active users, median activity was **1.00 conversation per user per day** (mean 1.37 ± 1.14; IQR 0.53). When restricted to Monday–Friday, the median remained **1.00 conversation per user per workday**, but the **mean increased to 1.51** ± **1.43** (IQR 1.00), indicating higher intensity of use on workdays. Usage was strongly workday-centric, with 88.7% of conversations occurring on weekdays, peaking early in the week and tapering toward weekends. Percentile distributions indicate broad but generally light adoption with a pronounced long tail of power users (Table 2).

**Table 2:** Per-user conversation rates by percentile (all users). Values are conversations per user per day; workdays restrict to Mon–Fri.

	10^th^	25^th^	50^th^	75^th^	90^th^
All days	0.506	1.000	1.000	1.500	2.000
Workdays	0.750	1.000	1.000	2.000	2.500

**Fig 5:**
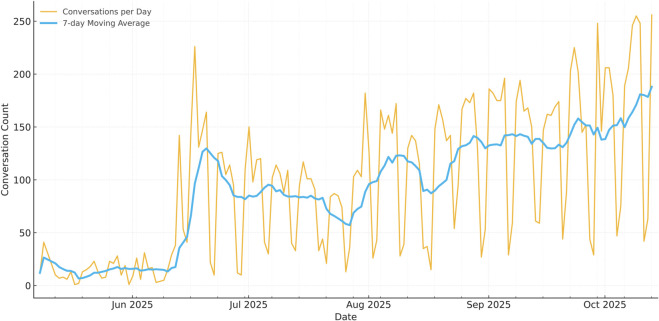
Conversations over time. Daily conversation counts with a 7-day moving average. The trajectory shows modest pilot usage, a validation-driven spike, and a subsequent gradual ascent, consistent with routine integration. Raw data available in S2 File.

As shown in Fig 5, daily volume progresses through three stages: low activity during the pilot, a short spike during pre-launch validation across services, followed by a steady upward trend consistent with organic adoption.

Conversation-level categorization indicates a task mix anchored in information access and documentation. The leading category was **specific information retrieval** (36.8%, 5,485/14,910), followed by **summarization** (26.5%, 3,954/14,910), **note writing** (20.7%, 3,086/14,910), and **clinical decision support** (11.0%, 1,643/14,910); the remainder (**other**, 5.0%, 742/14,910) encompassed translation, formatting, and miscellaneous requests. This mirrors the pilot and prior reports: clinicians primarily leverage LLM assistants to find and synthesize information rather than to outsource diagnostic reasoning.

Explicit feedback was sparse relative to interaction volume but balanced overall: **190 ratings** were recorded (**90 positive**, **100 negative**), representing <1% of assistant turns. This proportion is lower than in the pilot phase, suggesting that the earlier higher engagement likely reflected selection bias and a Hawthorne effect among volunteer participants. The reduction in feedback during routine deployment highlights a key limitation of relying solely on user-provided ratings for post-market monitoring. It raises concerns about the feasibility of continuously supervising such systems once embedded in clinical workflows. Complementary approaches such as systematic sampling of conversations or automated detection of potentially unsafe or clinically inconsistent responses by secondary models trained for this task may be necessary to ensure sustained oversight and safety at scale.

## Discussion

This work presents a fully on-premises, GDPR-compliant, EHR-embedded LLM-based assistant deployed in routine clinical care. During a one-month pilot involving 28 physicians from nine specialties, the system processed 482 conversations, with 64% of participants using it daily. The prevailing usage category was related to information retrieval and synthesis, indicating that clinicians primarily viewed the assistant as a documentation and information-access aid rather than a diagnostic tool, which accounted for only 25.1% of uses.

Following the pilot, the assistant was deployed hospital-wide and adopted by 1,028 users who generated 14,910 conversations over a five-month period. Of these, 561 users (54.6%) were active at least once per week. Usage concentrated on weekdays, with most interactions centered on information retrieval (36.8%) and summarization (26.5%), confirming that the assistant had become a stable and pragmatic support tool within daily clinical workflows rather than a transient novelty. This sustained engagement demonstrates that large-scale, on-premises deployment of LLM systems in clinical environments is feasible when integration, security, and governance are prioritized from the outset.

Despite these encouraging results, several limitations remain. The study is single-center, of short duration relative to long-term clinical adoption, and does not include quantitative analyses or objective workload measures such as time savings or after-hours charting. The reduction in explicit feedback compared with the pilot suggests that continuous human-based monitoring becomes difficult once the system is integrated into daily practice. Future work should explore alternative oversight mechanisms such as random sampling of interactions and automated detection of potentially unsafe or inconsistent model outputs.

Next steps include multi-site deployment, quantitative evaluation of workflow efficiency and clinician satisfaction, followed by iterative fine-tuning of domain-specific models. Overall, our findings show that secure, locally governed LLM systems can be adopted at scale and demonstrate practical utility for clinical documentation and information access, providing a replicable model for responsible AI integration in healthcare.

## Supporting information

S1 Appendix Technical details. Additional information on the implementation of PubMed data extraction and loading, and processing of eHealth Network data.
(PDF)

S1 File Conversation classification script. Python script used for automatic classification of the user’s initial message.
(PY)

S2 File Anonymized usage logs. Logs of conversation creation activity, including anonymized user identifiers and creation timestamps.
(CSV)
