# Supplementary material for: Implementation of large language models in electronic health records
Source: PLOS Digit Health. 2025 Dec 19;4(12):e0001141. doi: 10.1371/journal.pdig.0001141 (PMC12716761; doi:10.1371/journal.pdig.0001141)
Supplement: S1 Appendix — Additional information on the implementation of PubMed data extraction and loading, and processing of eHealth Network data. (PDF) [file pdig.0001141.s001.pdf]

TECHNICAL DETAILS

A. PUBMED

In practice, PubMed exposes an FTP service that contains a structured representation of articles and eBooks, which we use to download the selected subset of articles and data relevant to our physicians. The documents are then parsed and transformed into Markdown before being indexed in Apache Solr.

B. EHEALTH NETWORK

Fig. 1 outlines the retrieval pipeline and its caching layer. To reduce Docling's load and improve latency (OCR on large PDFs can exceed a minute), we cache extracted documents for 30 days in Redis. The cache key concatenates the patient's identifier with the document's eHealth identifier, ensuring that a document can be retrieved only when both identifiers are known and preventing access to files deleted from the eHealth network or belonging to other patients.

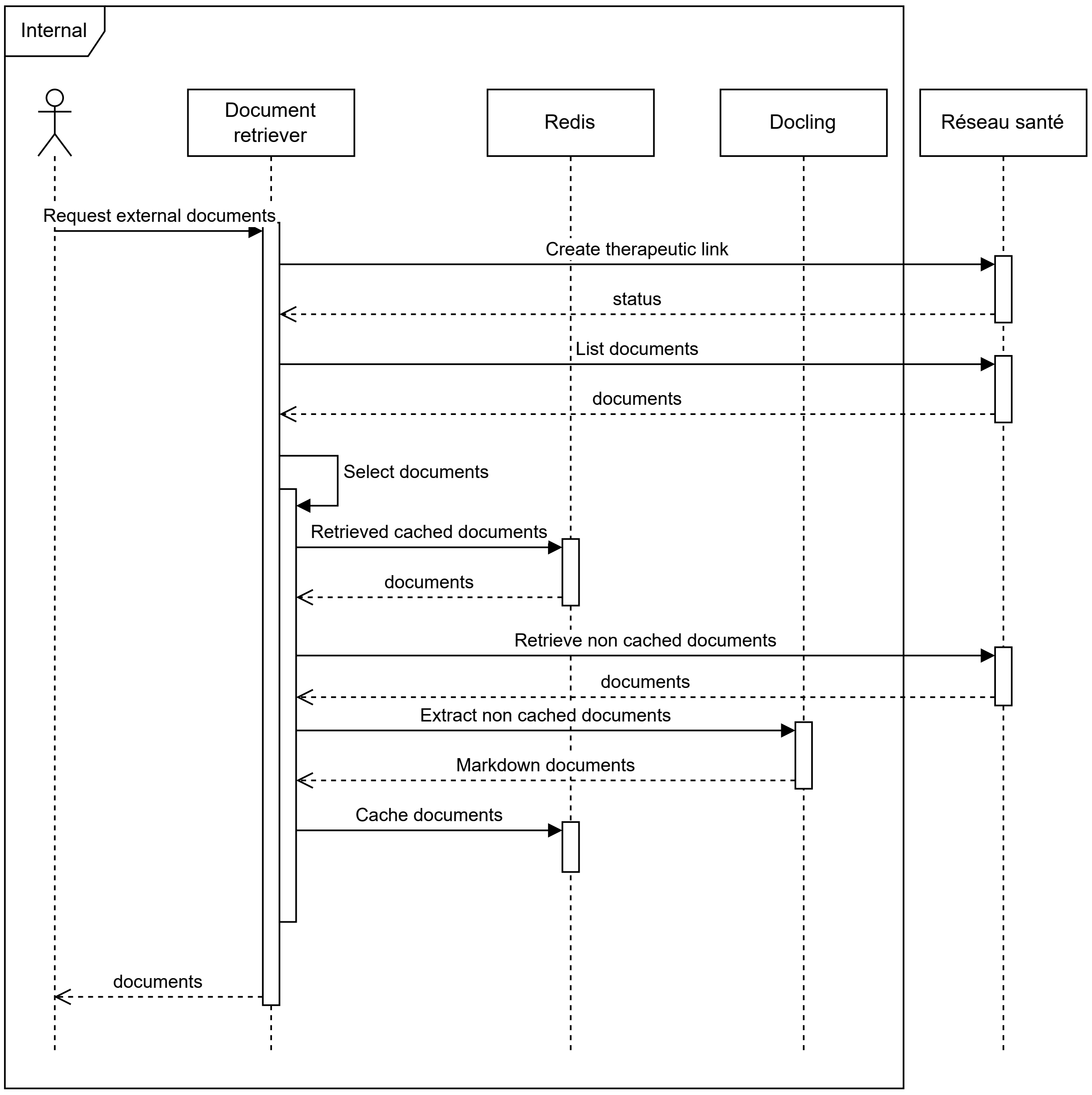

FIGURE 1. **External document retrieval and extraction process**
